# Bridging Human Resources for Health (HRH) gaps by applying clinical mentoring in selected health facilities: evidence from Jigawa State in northern Nigeria

**DOI:** 10.1186/1472-6963-14-S2-P89

**Published:** 2014-07-07

**Authors:** Ekechi Okereke, Jamilu Tukur, Jean Butera, Bello Mohammed, Ibrahim Yisa, Benson Obonyo, Amina Aminu, Mike Egboh

**Affiliations:** 1 Abt Associates Nigeria/Partnership for Transforming Health Systems Phase 2, Abuja, Federal Capital Territory, Nigeria; 2 Department of Obstetrics/Gynecology, Bayero University/Aminu Kano Teaching Hospital, Kano, Nigeria

## Background

In July 2012, a clinical mentoring intervention commenced in Jigawa State through collaboration between the Jigawa State Ministry of Health and the Partnership for Transforming Health Systems Phase 2 project. After 6 months, an evaluation was undertaken to assess whether clinical mentoring has benefits for the health workforce situation within the intervention health facilities as well as whether it improved maternal, newborn and child health service delivery in Jigawa State within northern Nigeria.

## Materials and methods

Multiple approaches to data collection were undertaken. Pretested interviewer-administered questionnaires were used to determine if there was an increase in the clinical knowledge levels of the mentored health workers after 6 months. Operational and service statistics of the intervention facilities were examined 6 months before the commencement of the clinical mentoring intervention as well as six months thereafter. Quantitative data was analyzed with SPSS version 20. In-depth interviews were undertaken with the clinical mentors and Jigawa State government health officials. Semi-structured interviews were undertaken with the mentored health workers and health facility departmental heads for Obstetrics and Pediatrics. Qualitative data was audio-recorded, transcribed and thematically analyzed.

## Results

Significant improvements in the professional capacity of mentored health workers were observed by clinical mentors, heads of departments and the mentored health workers. Across five health facilities, over 90% of the 33 mentored health workers recorded increases in their knowledge test scores after a 6 months period suggesting an improvement in their clinical knowledge and skills. Maternal and newborn deaths decreased in three out of the five clinical mentoring health facilities while normal deliveries increased in two out of the five intervention health facilities. Best practices were introduced with the support of the clinical mentors such as appropriate baseline investigations for pediatric patients, the use of magnesium sulphate and misoprostol for the management of eclampsia and post-partum hemorrhage respectively as well as the use of ambu-bags for neonatal resuscitation to reduce neonatal mortality. Government health officials indicate that clinical mentoring has led to more emphasis on the need for health workers to provide better quality health care services.

## Conclusions

The study shows that clinical mentoring is beneficial for improving the knowledge and clinical skills of mentored health workers as well as improving health service statistics. The introduction of clinical mentoring into the Jigawa State health system has improved the capacity of the mentored health care workers to deliver better quality maternal, newborn and child health services.

